# Self-administered questionnaire assessing childhood cancer treatments and associated risks for adverse health outcomes - The KiKme study

**DOI:** 10.3389/fonc.2023.1150629

**Published:** 2023-04-14

**Authors:** Lara Kim Brackmann, Ronja Foraita, Heike Schwarz, Alicia Poplawski, Thomas Hankeln, Danuta Galetzka, Sebastian Zahnreich, Claudia Spix, Maria Blettner, Heinz Schmidberger, Manuela Marron

**Affiliations:** ^1^Epidemiological Methods and Etiological Research, Leibniz Institute for Prevention Research and Epidemiology – BIPS, Bremen, Germany; ^2^Faculty of Mathematics and Computer Science, University of Bremen, Bremen, Germany; ^3^Biometry and Data Management, Leibniz Institute for Prevention Research and Epidemiology – BIPS, Bremen, Germany; ^4^Institute of Medical Biostatistics, Epidemiology and Informatics, University Medical Center of the Johannes Gutenberg University, Mainz, Germany; ^5^Institute of Organismic and Molecular Evolution, Molecular Genetics and Genome Analysis, Johannes Gutenberg University, Mainz, Germany; ^6^Department of Radiation Oncology and Radiation Therapy, University Medical Center of the Johannes Gutenberg University, Mainz, Germany; ^7^German Childhood Cancer Registry, Division of Childhood Cancer Epidemiology (EpiKiK), Institute for Medical Biostatistics, Epidemiology and Informatics, University Medical Center of the Johannes Gutenberg University, Mainz, Germany

**Keywords:** childhood cancer survivors (CCS), second malignancies, radiotherapy, chemotherapy, body mass index - BMI, thyroid diseases, lipid metabolism, validation

## Abstract

**Background:**

Childhood cancer survivors (CCS) are at particularly high risk for therapy-related late sequelae, with secondary primary neoplasms (SPN) being the most detrimental. Since there is no standardized questionnaire for retrospective assessment of associations between prior cancer treatments and late health effects, we developed a self-administered questionnaire and validated it in a cohort of CCS.

**Methods:**

CCS of a first primary neoplasm (FPN, N=340) only or with a subsequent SPN (N=101) were asked whether they had received cancer therapies. Self-reports were compared to participants’ medical records on cancer therapies from hospitals and clinical studies (N=242). Cohen’s Kappa (κ) was used to measure their agreement and logistic regression was used to identify factors influencing the concordance. Associations between exposure to cancer therapies and late health effects (overweight/obesity, diseases of the lipid metabolism and the thyroid gland, cardiovascular diseases, occurrence of SPN) were analyzed in all participants by applying generalized linear mixed models to calculate odds ratios (OR) and 95% confidence intervals (95%CI).

**Results:**

For CCS of SPN, a perfect agreement was found between self-reports and medical records for chemotherapy (CT, κ=1.0) while the accordance for radiotherapy (RT) was lower but still substantial (κ=0.8). For the CCS of FPN the accordance was less precise (CT: κ=0.7, RT: κ=0.3). Cancer status, tumors of the central nervous system, sex, age at recruitment, vocational training, follow-up time, and comorbidities had no impact on agreement. CCS with exposure to CT were found to be less often overweight or obese compared to those without CT (OR=0.6 (95%CI 0.39; 0.91)). However, they were found to suffer more likely from thyroid diseases excluding thyroid cancers (OR=9.91 (95%CI 4.0; 24.57)) and hypercholesterolemia (OR=4.45 (95%CI 1.5; 13.23)). All other analyses did not show an association.

**Conclusion:**

Our new questionnaire proved reliable for retrospective assessment of exposure to CT and RT in CCS of SPN. For the CCS of FPN, self-reported RT was very imprecise and should not be used for further analyses. We revealed an association between late health outcomes occurring as hypercholesterolemia and thyroid diseases, excluding thyroid cancer, and the use of CT for the treatment of childhood cancer.

## 1 Background

Patients with childhood cancer are often treated with radiotherapy (RT) and/or chemotherapy (CT) (1). Over the last decades, these therapies for childhood cancer have improved significantly, which have been accompanied by improvements in long-term survival (2, 3). However, since these therapies not only affect the tumor but also healthy tissues, they are known factors associated with the development of second primary neoplasms after childhood cancer (4) or can result in several late adverse health effects (5). Despite similar therapies, not all childhood cancer survivors (CCS) suffer from long-term health effects. Data from 2018 showed that around 8% of the CCS listed in the German Childhood Cancer Registry develop a second primary neoplasm (SPN) within the next 35 years (6). In addition to this particularly serious late adverse health outcome after primary cancer treatment in childhood, CCS are at increased risk for chronic cardiovascular or lung diseases, as well as infertility (7–12). The risk of such late-occurring health issues seems to be associated with the dose of RT and CT (11, 13, 14).

In a large cohort of CCS it has been shown that a reduction in radiation exposure during therapy leads to fewer cardiac events in adulthood (2). In particular, irradiation of the mediastinum or spinal cord, for example in the context of treatment for Hodgkin’s lymphoma or tumors of the central nervous system (CNS), is considered as a risk factor for the development of cardiac disease later in life (15). Similarly, CCS are at increased risk of developing restrictive lung diseases after thoracic RT. Due to the formation of the lung alveoli in the first few years of life, exposure to ionizing radiation at this age is moreover associated with reduced lung capacity (15).

In the case of both RT and CT, the dose of therapy received is of importance for the development of heart and lung diseases later in life (16). While treatment with high doses of CT agents is associated with an increased risk of cardiac events (17), lower doses are associated primarily with conditions considered to be risk factors for the development of late cardiac events (18).

RT and CT are used not only as definitive treatments/monotherapy, but also as part of multimodal therapy strategies. A combination of RT and CT has become established as the standard treatment for many cancer sites (19), as the combination of systemically acting CT and RT often achieves better therapeutic results (20). In this combination, the systematically acting CT acts by a radiosensitization mechanism that involves making tumor cells more sensitive to RT (21, 22). Due to an additive or synergistic effect of this multimodal therapy, CCS are at increased risk for the development of late adverse health effects (e.g., cardiovascular, hematological, neurological, pulmonary, and renal conditions) compared to CCS treated with monotherapy (22).

In addition to RT and CT or the combination of these two therapeutic strategies, childhood cancers are now also treated with targeted and cancer-specific approaches. Immunologic and targeted therapies are increasingly finding their way into the treatment of pediatric cancers because, unlike CT and RT, they are cancer-specific and not genotoxic, and thus may reduce the risk of late effects (23).

Malignant diseases of the hematopoietic or lymphatic system, which occur particularly frequently in childhood, are often successfully treated with hematopoietic stem cell transplantation (23, 24). In order to prevent rejection reactions such as graft-versus-host diseases after transplantation, post-transplant treatments with immunosuppressants are required. Due to this combination of therapies, recipients of stem cell transplants have a further increased risk of late adverse health effects such as diseases of the kidney and liver, development of SPN, as well as overall reduced quality of life (24).

Despite the important role of cancer therapies in the development of late adverse health effects after surviving childhood cancer, research on accurate exposure measures of cancer therapies in childhood often remains challenging due to a lack of valid information on cancer therapies in many epidemiologic studies. To our knowledge, the only attempt of asking young adults about their exposure to cancer therapies in childhood was done *via* telephone interviews within the Childhood Cancer Survivor Study in the early 2000s (25). However, until now, there is no established self-administered questionnaire to retrospectively assess exposures to RT, CT, or other cancer therapies as well as to diagnostic procedures in childhood among a population of young adults. Particularly in countries where the linkage of different medical data (e.g., from hospitals, outpatient care, registries, and health insurance companies) has so far only been possible to a limited extent and often only at great expense for reasons of data protection (26) or infrastructural issues, such a questionnaire would be of great benefit to be able to ask study participants in an uncomplicated way for information on cancer therapies received. Therefore, a new self-administered questionnaire, which consists of a total of 62 items in total, was developed and applied within the population of the nested case-control study KiKme (27). Nine of the questionnaire items collect detailed information about lifetime medical exposures to radiation and cancer therapies.

To validate this new questionnaire, this study aims, first, to compare self-reported exposure to cancer therapies with information on cancer treatment from medical records. Therefore, a subsample of study participants with complete information from both questionnaires and medical records from hospitals and therapy-optimizing studies will be used. Secondly, influencing factors on concordance between the questionnaire and medical records will be analyzed. Finally, reliable self-reported information from our questionnaire will be used to estimate possible associations of exposure to cancer therapies with the risk of late adverse health effects within the KiKme study population.

## 2 Materials and methods

### 2.1 Study design and participants

The study participants were recruited within the population-based nested case-control study KiKme. Detailed information on recruiting strategies and data collection can be found in the study protocol (27). In brief, the KiKme study population included 441 CCS, registered at the German Childhood Cancer Registry. CCS were grouped into survivors with a first primary neoplasm (FPN, n=340) only and survivors with a subsequent SPN (n=101). CCS with FPN only were used as cancer controls and were matched to participating CCS with an SPN by age, sex, cancer site, year of diagnosis, and age at diagnosis. In addition, the study population includes 150 cancer-free controls that were recruited at the Department of Orthopedics and Trauma Surgery at the Johannes Gutenberg-University in Mainz (Germany). Cancer-free controls were matched by sex and age to the SPN and FPN survivors.

### 2.2 Data collection

The data collection in the study was done using our newly developed questionnaire which was self-administered by all participants. In 62 questions the study participants were asked to provide information about their demographics, health and health-related behaviors, regular medication, as well as severe diseases in their families. The study participants were also allowed to obtain information from others, e.g., their parents, in order to answer the questionnaire.

Based on anthropometric information on weight and height, normal weight was defined as Body Mass Index (BMI) of 18.5 and < 25 kg/m^2^, overweight as BMI ≥25 kg/m^2^, and obesity as BMI ≥30 kg/m^2^ according to the WHO standards. To assess their medical history and health status, participants were asked whether they had been diagnosed with any severe disease, including hypercholesterolemia, hypertension, heart attack, stroke, and thyroid diseases. Additionally, age at diagnosis was requested.

Besides the questions on anthropometric factors and health, the questionnaire included nine questions on medical therapies and lifetime exposure to ionizing radiation. Within these nine questions participants were asked whether they had ever received cancer therapy (RT, CT, or other cancer therapy). If so, they were asked in what year, at what age, how often, and with what doses they were treated. Also, information on affected body regions and substances was collected. They were asked whether they had ever had diagnostic or interventional exposures, including radiographic examination, such as for fractures, pneumonia, surgery, or dental examinations, computed tomography, positron and single photon emission computed tomography, magnetic resonance imaging, minimally invasive radiological intervention, and thyroid radioiodine therapy.

To validate the information from the questionnaire, we used available data from medical records on cancer therapies recorded by treating hospitals or therapy-optimizing studies from a subsample of our participants and compared them with participants’ self-reported information.

### 2.3 Statistical *analysis*

Descriptive analyses were performed on age, sex, cancer diagnoses, subsequent therapies, and exposure to medical diagnostics. Results were stratified by cancer status (SPN, FPN, and cancer-free controls) and frequency (N) and proportions (%) were provided for summary statistics.

A quality assessment was performed to determine the validity and agreement of self-reported information on therapy (received RT/CT: yes/no) with the information from the medical records. This was measured by Cohen’s kappa coefficient (κ) (28). Influencing factors (sex, age, number of neoplasms, tumors of the CNS, vocational training, comorbidities, time since cancer treatment) on the concordance between the questionnaire and medical records were analyzed using logistic regression.

We applied generalized linear mixed models (GLMM) to the self-reported information from questionnaires to analyze the statistical association between exposure to cancer therapies and risk of later occurring adverse health effects as well as to calculate odds ratios (OR) and 95% confidence intervals (95%CI). For the analysis of adverse health effects after exposure to cancer therapies, CCS of FPN and CCS of SPN were aggregated to ensure a sufficiently large cell population for each adverse health effect occurring after the FPN and prior to a possible SPN. For CCS of SPN, only therapies for the FPN were included in analyses. For the analysis of the occurrence of an SPN later in life, cancer-free control patients were excluded. In our models, each matching group was treated as a random effect. Additionally, ‘age’ and ‘year of birth’ were included as fixed effects in all models to improve matching efficiency for the variable ‘age at recruitment’ within the specified 5-year period (27). Possible additional adjustment variables were considered after drawing a Directed Acyclic Graph (DAG) that was carefully developed based on prior knowledge using DAGitty (version 3.0)^1^ (see **Supplementary File 1**).

Survival analysis using Kaplan-Meier curves was applied to describe and compare the cumulative incidence curves of the onset of late adverse health effects by cancer site (leukemia, lymphoma, and tumors of the central nervous system) and stratified by self-reported cancer therapy. For this purpose, the year of diagnosis of the late adverse disease was subtracted from the year of reported therapy. All statistical analyses for this publication were performed using SAS 9.3 (SAS Institute Inc., Cary, North Carolina, USA).

## 3 Results

### 3.1 Study characteristics

This study includes 591 participants of which 51% were females (**Table 1**). The mean age at the interview was 35.14 years (standard deviation (SD): 7.14; range: 19.90-51.40 years) for CCS of SPN, 34.84 years (SD: 7.68; range: 19.60-54.50 years) for CCS of FPN, and 28.91 years (SD: 7.32; range: 18.70-48.20 years) for cancer-free controls. The interviews were conducted on average 27.26 years (SD: 6.90; range 5.0-38.0 years) after the first cancer diagnosis in CCS of SPN and 26.24 years (SD: 6.93; range: 4-39 years) after the first diagnosis in CCS of FPN. Leukemia and lymphoma were most commonly treated with both RT and CT in our study (leukemia: N=105, 50%, lymphoma: N=85, 47%, **Supplementary Figures 1A, B**), regardless of the chronological order of the two therapies. For tumors of the CNS either RT and CT or a combination with an additional therapy (e.g., stem cell transplantation) was most likely (N=17, 29% for both, **Supplementary Figure 1C**). Further characteristics of the study participants including detailed information on cancer diagnoses and treatment as well as on exposure to medical diagnostics are summarized in **Table 1**. Participants who did not provide information in self-administered questionnaires (N=37, 6%) were excluded from the analyses. For 272 (46%, 93 CCS of SPN and 179 CCS of FPN) of the KiKme study participants information was available from medical records (**Table 2**). Of these participants, 235 (86%) had received RT or CT, five (2%) participants had only undergone a stem cell transplant, and for another two (1%) participants no therapy was indicated. For the remaining 30 (11%) study participants, information on cancer therapies from medical records was not available.

**Table 1:** Description of the study population.

	CCS of SPN (N=101)		CCS of FPN (N=340)		CO (N=150)		Total (N=591)	
	n	%	n	%	n	%	n	%
Questionnaire available								
Yes	85	84%	325	96%	144	96%	554	94%
No	16	16%	15	4%	6	4%	37	6%
Sex								
Female	50	50%	189	56%	62	41%	301	51%
Male	51	50%	151	44%	88	59%	290	49%
Age at interview								
< 25 years	9	9%	37	11%	54	36%	100	17%
25-29 years	14	14%	55	16%	37	25%	106	18%
30-34 years	17	17%	76	22%	20	13%	113	19%
35-39 years	21	21%	70	21%	20	13%	111	19%
≥ 40 years	24	24%	87	26%	13	9%	124	21%
Cancer site of FPN								
Leukemia	41	41%	166	49%	–	–	207	35%
Lymphoma	41	41%	135	40%	–	–	176	30%
Brain & CNS	15	15%	35	10%	–	–	50	8%
Other tumors	4	4%	4	1%	–	–	8	1%
Cancer site of SPN								
Thyroid cancer	30	30%	–	–	–	–	30	5%
Skin carcinoma	32	32%	–	–	–	–	32	5%
Malignant melanoma	4	4%	–	–	–	–	4	1%
Leukemia	9	9%	–	–	–	–	9	2%
Lymphoma	6	6%	–	–	–	–	6	1%
Brain & CNS	9	9%	–	–	–	–	9	2%
Breast cancer	3	3%	–	–	–	–	3	1%
Other unspecific carcinoma	7	7%	–	–	–	–	7	1%
Sarcoma	2	2%	–	–	–	–	2	0%
Radiotherapy								
Ever	68	67%	222	65%	3	2%	293	50%
For FPN diagnosis	62	61%	215	63%	–	–	277	47%
For SPN diagnosis	7	7%	2	1%	–	–	9	2%
For other diseases	0	0%	0	0%	3	2%	3	1%
Never	15	15%	95	28%	140	93%	250	42%
No information	18	18%	23	7%	7	5%	48	8%
Chemotherapy								
Ever	82	81%	314	92%	0	0%	396	67%
For FPN diagnosis	78	77%	314	92%	–	–	392	66%
For SPN diagnosis	14	14%	0	0%	–	–	14	2%
Never	2	2%	7	2%	114	76%	123	21%
No information	17	17%	19	6%	36	24%	72	12%
Other cancer therapies								
Ever	59	58%	79	23%	0	0%	138	23%
For FPN diagnosis	17	17%	66	19%	–	–	83	14%
*Surgery*	*17*	*17%*	*56*	*16%*	*-*	*-*	*73*	*12%*
*Other cancer therapies^1^*	*2*	*2%*	*9*	*3%*	*-*	*-*	*11*	*2%*
For SPN diagnosis	52	51%	4	1%	–	–	56	9%
*Surgery*	*47*	*47%*	*4*	*1%*	*-*	*-*	*51*	*9%*
*Other cancer therapies^1^*	*2*	*2%*	*0*	*0%*	*-*	*-*	*2*	*0%*
For further cancer diagnosis	5	5%	0	0%	–	–	5	1%
*Surgery*	*3*	*3%*	*0*	*0%*	*-*	*-*	*3*	*1%*
Never	23	23%	226	66%	113	75%	362	61%
No information	19	19%	35	10%	37	25%	91	15%
X-ray examinations								
Ever	84	83%	310	91%	141	94%	535	91%
Never	0	0%	5	1%	3	2%	8	1%
No information	17	17%	25	7%	6	4%	48	8%
Computed tomography examinations								
Ever	73	72%	237	70%	68	45%	378	64%
Never	6	6%	56	16%	67	45%	129	22%
No information	22	22%	47	14%	15	10%	84	14%
Positron emission tomography								
Ever	29	29%	69	20%	0	0%	98	17%
Never	32	32%	186	55%	138	92%	356	60%
No information	40	40%	85	25%	12	8%	137	23%
Magnetic resonance imaging								
Ever	75	74%	229	67%	109	73%	413	70%
Never	5	5%	56	16%	32	21%	93	16%
No information	21	21%	55	16%	9	6%	85	14%
Minimally invasive radiological intervention								
Ever	13	13%	30	9%	7	5%	50	8%
Never	58	57%	261	77%	142	95%	461	78%
No information	30	30%	49	14%	1	1%	80	14%
Thyroid radioiodine therapy								
Ever	22	22%	26	8%	1	1%	49	8%
Never	58	57%	261	77%	142	95%	461	78%
No information	21	21%	53	16%	7	5%	81	14%
Weight status								
Underweight (BMI < 18.5 kg/m²)	2	2%	11	3%	3	2%	16	3%
Normal weight (BMI ≥ 18.5 - < 25 kg/m²)	45	45%	167	49%	69	46%	281	48%
Overweight (BMI ≥ 25 - < 30 kg/m²)	28	28%	93	27%	47	31%	168	28%
Obesity (BMI ≥ 30 kg/m²)	9	9%	50	15%	25	17%	84	14%
No information	17	17%	19	6%	6	4%	42	7%
Thyroid diseases (without cancer)^2^								
Yes	18	18%	117	34%	5	3%	140	24%
No	42	42%	208	61%	134	89%	384	65%
No information	41	41%	15	4%	11	7%	67	11%
Hypercholesterolemia								
Yes	15	15%	48	14%	3	2%	66	11%
No	67	66%	274	81%	134	89%	475	80%
No information	19	19%	18	5%	13	9%	50	8%
Cardiovascular diseases^3^								
Yes	13	13%	46	14%	10	7%	69	12%
No	69	68%	271	80%	129	86%	469	79%
No information	19	19%	23	7%	11	7%	53	9%

BMI, body mass index; CCS, childhood cancer survivors; CO, cancer-free control; FPN, first primary neoplasm; SPN, second primary neoplasm.

^1^Other cancer therapies include stem cell transplantation and other medication.

^2^Malignant thyroid diseases were considered as SPN.

^3^Including hypertension, heart attack, or stroke.

**Table 2:** Available therapy information from medical records of KiKme study participants.

	CCS of SPN (N=93)		CCS of FPN (N=179)		Total (N=272)	
	n	%	n	%	n	%
Radio- and/or chemotherapy	86	92%	149	83%	235	86%
Only radiotherapy	1	1%	3	2%	4	1%
Only chemotherapy	20	22%	31	17%	51	19%
Radiochemotherapy	65	70%	115	64%	180	66%
Stem cell transplantation^1^	10	11%	9	5%	19	7%
Only stem cell transplantation	0	0%	5	3%	5	2%
Stem cell transplantation with radio-/chemotherapy	10	11%	4	2%	14	5%
No therapy	2	2%	0	0%	2	1%
Missing data	5	5%	25	14%	30	11%

CCS, childhood cancer survivors; FPN, first primary neoplasm; SPN, second primary neoplasm.

^1^Information on stem cell transplantation was not actively collected, available data are incidental findings. The actual number of transplantations is probably higher.

### 3.2 Association between self-reported cancer therapies and medical records

A perfect agreement (κ=1.0) was found between self-reports on CT from CCS of SPN and their corresponding information from medical records (**Table 3**). Overall, 71 (97%) CCS of SPN reported receiving CT and only two (3%) reported not receiving CT. The agreement for RT was lower but substantial (κ=0.77). Three (5%) CCS of SPN misreported on RT, while there was an agreement between both data sources for the remaining 59 (95%) CCS of SPN. Overall, the group of CCS of FPN reported less accurately. For CT, a moderate agreement was observed (κ=0.66). However, only one (1%) CCS of FPN misreported by indicating no CT in the self-reported questionnaire whereas there was information on CT available in the medical records. The other 140 (99%) CCS of SPN with available information on CT reported correctly. The lowest and only fair agreement was found for RT in CCS of FPN (κ=0.31). Whereas 105 (93%) CCS of FPN reported correctly on RT, a total of eight (7%) CCS of FPN reported that they did not receive RT while RT was documented in their medical records. Using logistic regression, none of the variables (cancer status, sex, age at recruitment, tumors of the CNS, vocational training, follow-up time, and comorbidities) had significant impact on the agreement between self-reported or clinically documented RT (**Table 4**).

**Table 3:** Concordance between self-reported exposure to cancer therapies and data from medical records.

	Information from medical records											
	CCS of SPN				CCS of FPN				Total			
	*Received chemotherapy*		*Received radiation therapy*		*Received chemotherapy*		*Received radiation therapy*		*Received chemotherapy*		*Received radiation therapy*	
	Yes	No	Yes	No	Yes	No	Yes	No	Yes	No	Yes	No
Questionnaire data												
*Received therapy*												
Yes	71	0	53	2	139	0	103	0	210	0	156	2
No	0	2	1	6	1	1	8	2	1	3	9	8
*K*	1.00		0.77		0.66		0.31		0.85		0.56	

CCS, childhood cancer survivors; FPN, first primary neoplasm; SPN, second primary neoplasm.

**Table 4:** Influencing factors on the correlation between self-reported exposure to radiotherapy and data from medical records of participants from the KiKme study.

	Total (n=175)		Not concordant (n=11)		Concordant (n=164)		OR (95% CI)
	n	%	n	%	n	%	
Second primary neoplasm							
No	113	65%	8	73%	105	64%	Ref.
Yes	62	35%	3	27%	59	36%	1.08 (0.25; 4.75)
Sex							
Female	102	58%	9	82%	93	57%	Ref.
Male	73	42%	2	18%	71	43%	2.43 (0.46; 12.88)
Age							
< 35 years	74	42%	5	45%	69	42%	Ref.
≥ 35 years	101	58%	6	55%	95	58%	1.14 (0.16; 7.97)
Tumors of the CNS							
No	152	87%	10	91%	142	87%	Ref.
Yes	23	13%	1	9%	22	13%	1.35 (0.13; 13.99)
Vocational training							
Non-academic	98	56%	6	55%	92	56%	Ref.
Academic	64	37%	3	27%	61	37%	1.26 (0.29; 5.43)
Missing	13	7%	2	18%	11	7%	
Follow-up time							
< 25 years	67	38%	5	45%	62	38%	Ref.
≥ 25 years	108	62%	6	55%	102	62%	2.24 (0.34; 14.54)
Comorbidities^1^							
No	39	22%	2	18%	37	25%	Ref.
Yes	136	78%	9	82%	127	75%	1.39 (0.27; 7.34)

CI, confidence interval; CNS, central nervous system; OR, odds ratio.

^1^Comorbidities included diabetes, hypercholesterolemia, hypertension, lung diseases such as asthma or bronchitis, hay fever, inflammatory joint or vertebral diseases including arthrosis and rheumatism, neurodermatitis, heart attack, stroke, thyroid diseases, Epstein-Barr virus infections, HIV, Hepatitis, or any other severe disease.

### 3.3 Exposure to cancer therapies and risk of later occurring adverse health effects

Since CCS provided valid self-reports of CT, we analyzed potential associations of this treatment with adverse health effects. The results showed that CCS treated with CT were found to be less often overweight or obese compared to CCS without CT (OR=0.6 (95%CI 0.39; 0.91), **Table 5**). A total of 140 (24%) of the study participants reported on diseases of the thyroid gland (**Table 1**). Here, only non-malignant diseases were considered as thyroid diseases and malignant diseases of the thyroid gland were considered as SPN. Thyroid diseases occurred more often in participants with CT (OR=9.91 (95% CI 4.0; 24.57), **Table 5**). Similar results were obtained for hypercholesterolemia: Participants treated with CT were found to suffer more likely from such disorders of lipid metabolism (OR=4.45 (95%CI 1.5; 13.23)). No difference was found for the occurrence of cardiovascular diseases (OR=1.46 (95%CI 0.71; 3.01)) and second primary neoplasms (OR=0.28 (95%CI 0.05; 1.47)). The association between exposure to RT and late adverse health effects in the group of CCS of SPN could not be calculated due to small sample sizes (**Table 6**).

**Table 5:** Self-reported exposure to chemotherapy and risk of later adverse health effects in participants of the KiKme case-control study.

	n	%	n	%	n	%	OR (95% CI)
Weight status							
	Total (N=538)		Underweight or normal weight (N=292)		Overweight or obese (N=246)		Overweight/obesity vs. underweight/normal weight
Chemotherapy	388	72%	215	74%	173	70%	0.60 (0.39; 0.91)
No chemotherapy	150	28%	77	26%	73	30%	Ref.
Thyroid diseases (without cancer)							
	Total (N=497)		Yes (N=117)		No (N=380)		Yes vs. no
Chemotherapy	354	71%	111	95%	243	64%	9.91 (4.00; 24.57)
No chemotherap	143	29%	6	5%	137	36%	Ref.
Hypercholesterolemia							
	Total (N=520)		Yes (N=56)		No (N=464)		Yes vs. no
Chemotherapy	377	73%	52	93%	325	70%	4.45 (1.50; 13.23)
No chemotherapy	143	28%	4	7%	139	30%	Ref.
Cardiovascular diseases							
	Total (N=519)		Yes (N=60)		No (N=459)		Yes vs. no
Chemotherapy	374	72%	49	82%	325	71%	1.46 (0.71; 3.01)
No chemotherapy	145	28%	11	18%	134	29%	Ref.
Second primary neoplasm^1^							
	Total (N=398)		Yes (N=81)		No (N=317)		Yes vs. no
Chemotherapy	392	98%	78	96%	314	99%	0.28 (0.05; 1.47)
No chemotherapy	6	2%	3	4%	3	1%	Ref.

All analyses were adjusted for the matching group, birth year, and age at the interview. For CCS of SPN only chemotherapy for the first primary neoplasm was included in analyses. Participants with missing information were excluded from analysis.

CCS, childhood cancer survivors; CI, confidence interval; OR, odds ratio; SPN, second primary neoplasm.

^1^Cancer-free control patients were excluded from this analysis.

**Table 6:** Self-reported exposure to radiotherapy and late adverse health effects in CCS of SPN from the KiKme case-control study.

	n	%	n	%	n	%
Weight status						
	Total (N=80)		Underweight or normal weight (N=46)		Overweight or obese (N=34)	
Radiotherapy	62	78%	36	78%	26	76%
No radiotherapy	18	23%	10	22%	8	24%
Thyroid diseases (without cancer)						
	Total (N=54)		Yes (N=15)		No (N=39)	
Radiotherapy	38	70%	15	100%	23	59%
No radiotherapy	16	30%	0	0%	16	41%
Hypercholesterolemia						
	Total (N=73)		Yes (N=9)		No (N=64)	
Radiotherapy	56	77%	9	100%	47	73%
No radiotherapy	17	23%	0	0%	17	27%
Cadiovascular diseases						
	Total (N=76)		Yes (N=9)		No (N=67)	
Radiotherapy	58	76%	6	67%	52	78%
No radiotherapy	18	24%	3	33%	15	22%

For CCS of SPN only radiotherapy for the first primary neoplasm was included in analyses. Participants with missing information were excluded.

CCS, childhood cancer survivors; SPN, second primary neoplasm.

**Figure 1:**
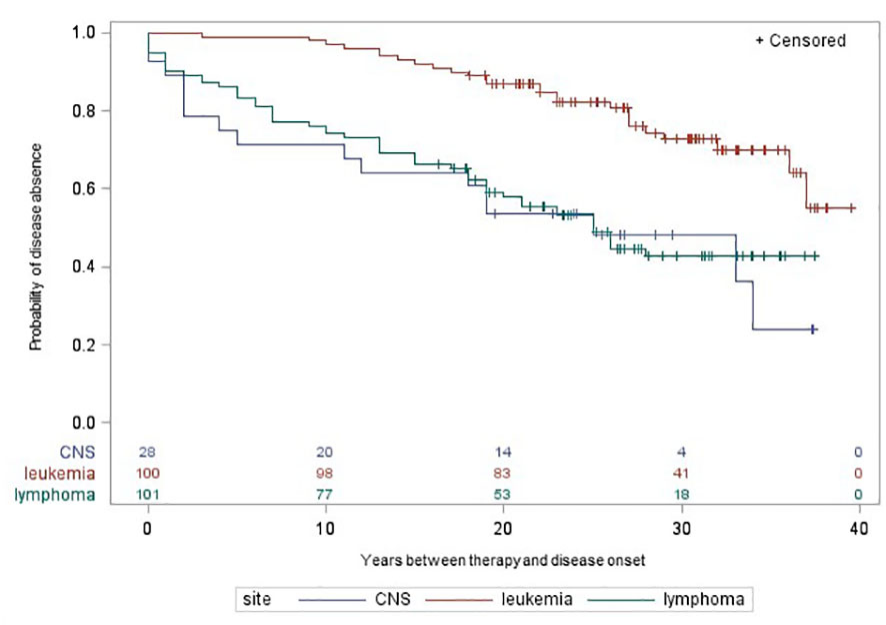
Time between self-reported radiotherapy and onset of late adverse diseases of the thyroid gland by cancer site in participants of the nested case-control study KiKme. Participants were included, if they received radiotherapy for a first primary cancer diagnosis (n=277). Thyroid cancers occurring as second primary neoplasms were excluded for this analysis. CNS, central nervous system.

**Figure 2:**
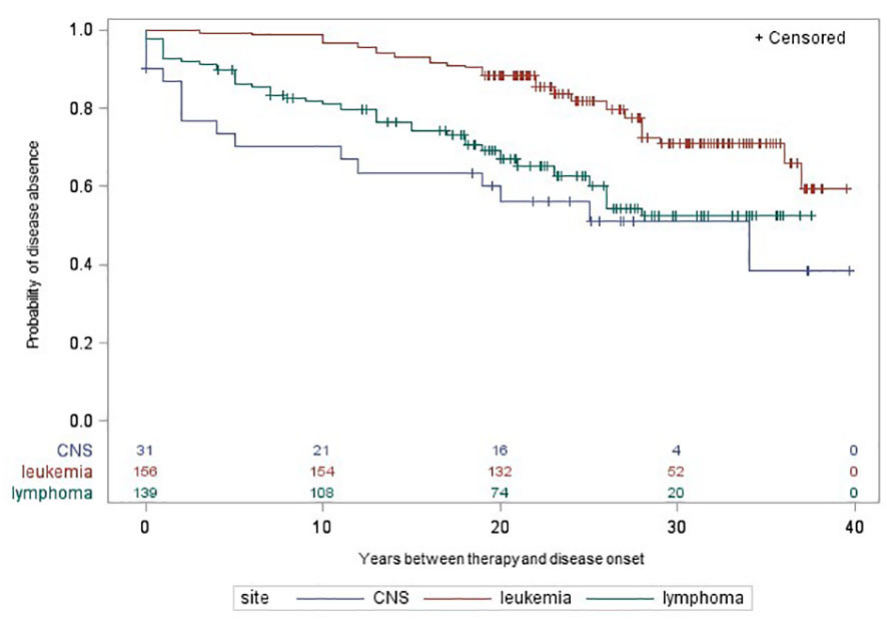
Time between self-reported chemotherapy and onset of late adverse diseases of the thyroid gland by cancer site in participants of the nested case-control study KiKme. Participants were included, if they received radiotherapy for a first primary cancer diagnosis (n=392). Thyroid cancers occurring as second primary neoplasms were excluded for this analysis. CNS, central nervous system.

397 CCS with RT and/or CT were included in the Kaplan-Meier analysis. **Figures 1**, **2** illustrate the cumulative incidence curves for late adverse diseases of the thyroid gland (excluding thyroid cancer) after exposure to cancer therapy for leukemia, lymphoma, and CNS tumors. The median follow-up time was 26.48 years (SD: 6.84 years, range: 4.0-36.0 years). After RT or CT, 26 (25%) or 38 (23%) CCS of leukemia, 54 (52%) or 56 (39%) CCS of lymphoma and 16 (52%) or 16 (47%) CCS of CNS tumors developed a non-malignant thyroid disease, respectively. The 20-year disease-free survival after primary cancer diagnosis was 54%, 64% in the group of leukemia CCS, 52% in CCS of lymphoma and 37% in CCS of CNS tumors. There were no remarkable differences between cancer sites in long-time survival for the other late adverse health outcomes.

## 4 Discussion

This study was successful in the validation of the newly developed self-administered questionnaire on the retrospective assessment of exposure to cancer therapies in childhood, especially regarding CT. Based on the data collected in this way, we demonstrated an impact of CT on health-related late effects in the cohort of CCS of the KiKme study. CCS with CT in childhood were found to suffer more likely from diseases of the thyroid gland and lipid metabolism. They were also less likely to be overweight or obese compared to CCS without CT. Self-reporting of RT in childhood was too imprecise to investigate associations with potential late effects.

### 4.1 Agreement between self-reported exposure and medical records

Similar analyses on the agreement between self-reported cancer therapy and medical records were previously conducted on survivors of adult breast cancer (29–35) or several other tumor entities (25, 32, 36).

The studies from other research groups showed good to very good agreements between self-reported exposure to CT and data from medical records. However, the follow-up period of the other studies was rather short. A study on breast cancer survivors by Kool et al. (35) found a high agreement for exposure to CT (κ=0.95) in a sample of 350 study participants after a short follow-up of 9 to 18 months after tumor surgery. An even shorter follow-up period was found in the study by Gupta and colleagues (34), where breast cancer survivors were asked about their disease and therapy approximately 6.5 months after their diagnosis. Considering that CT starts about 1 month after diagnosis and lasts for about one month, the time between last CT and interview was only about 4.5 months. The authors found moderate to excellent agreement for CT (81.7-98.0%). Besides the short time span between therapy and interview, patients in this study were provided with detailed information about their disease and therapy when they are discharged from the hospital, which might have contributed to the good agreement. In our study, study participants were asked about their exposure to CT about 27 years after the first cancer diagnosis in childhood. Nonetheless, we found similar rates of agreement for CT using our new developed questionnaire. The generally high compliance with CT might be attributed to recollection of the severe acute side effects of this treatment. This was also assumed in a recent review of self-reported medication in cancer survivors from Brüne et al. (32).

Contrary to the good agreement for CT, the self-reported exposure to RT was less precise in our study. Similarly, Gupta et al. (34) also report poor agreement with respect to RT. While 32.1% of participants reported RT, it was applied in only 4.9% of cases based on their medical records. Gupta et al. justify this phenomenon with the fact that RT is not used as first-line therapy in the curative treatment of breast cancer. Because RT is only used as a palliative or second/third-line therapy when surgery and CT were not able to control tumor growth or metastatic spread at a later time after diagnosis, it may not have been as well remembered by participants as CT. In contrast to our results and the results from Gupta et al., the study on breast cancer survivors by Kool et al. (35) found a high agreement for exposure to RT (κ=0.94) 9 to 18 months after tumor surgery. In addition, Roberts et al. (36) also reported a high agreement for exposure to RT (92%). However, they only investigated pelvic RT in the course of impact of cancer treatments on fertility in 101 young adult female cancer survivors. One possible cause for the differences in agreement regarding RT between the study of Roberts et al. and our study could be their underlying research question and the associated study population. They examined the impact of cancer and cancer treatments on reproductive health. In this context, pelvic RT as a potential cause of infertility might be particularly remembered by the respondents.

However, with the exception of the study by Roberts et al. (36), which includes survivors that were diagnosed during childhood or early adulthood, all other beforementioned validation studies have been conducted in adults. To the best of our knowledge, the only other study that ever requested information from young adults about their exposure to cancer therapies in childhood was the Childhood Cancer Survivor Study (25). In the early 2000s they completed telephone interviews and compared information from their study participants to therapy information assessed at the baseline survey. In total, they found a high agreement for exposure to CT (94%) and RT (89%) in their survey. In their validation study, participants who received input from others during interviews were excluded. In contrast, we gave our self-administered questionnaires to our study participants and gave them as much time as they needed to fill them out. They were also allowed to gather as much information from others as they wanted. Likely, many of the survivors who suffered from cancer in their early childhood cannot remember the therapy exactly when they are adults (25). In contrast, memory is likely to be very present in relatives, especially parents, for many years after therapy. To obtain the most accurate information possible, we encouraged our participants to also obtain information from others (e.g., from parents). In this way, we were able to retrospectively collect particularly accurate information about cancer therapies in early childhood.

Regarding factors that may have an influence on the agreement between self-reports and medical records, none of the chosen variables in our study (cancer status, sex, age at recruitment, tumors of the CNS, vocational training, follow-up time, and comorbidities) were found to be associated with the agreement. Also, Kool and colleagues investigated factors influencing concordance. In line with our findings, age had no significant impact on agreement for RT and CT in their study. Moreover, they could not demonstrate any influence of CT and endocrine therapy (35). In addition, in three studies (29, 30, 33) included in the review by Brüne et al. (32) neither age nor education had a significant effect on agreement regarding CT. Only the group by Roberts et al. (36) found significant associations between agreement and age as well as cancer recurrence. Here, younger age at diagnoses and cancer recurrence was associated with a higher risk of misreporting. These identified influencing factors seem reasonable to us since memory may not be as good for diagnoses at a younger age and therapies may have been mixed up by participants with multiple diagnoses. By encouraging our study participants to obtain information from others, we seemed to be able to successfully circumvent this effect in our study.

### 4.2 Late adverse health effects after cancer therapy

Previously, we investigated associations between cancer status and the occurrence of tumor therapy-related late adverse health effects in CCS of the KiKme study (37). In these analyses, however, cancer therapies were only considered as potential confounders. We found associations between cancer status and individual diseases including body mass index, hypercholesterolemia, and thyroid diseases excluding thyroid cancer. In detail, we observed that CCS of FPN and SPN were less likely to be overweight or obese than cancer-free controls. In an analysis of individual diseases, it was found that CCS suffer more frequently from thyroid diseases other than thyroid cancer and hypercholesterolemia compared to controls. Since these strong effects were only observed when comparing CCS to cancer-free controls and disappeared when comparing CCS of SPN to CCS of FPN, we hypothesized that the effect may be driven by cancer therapies and conducted the present study.

Our current analyses show that thyroid disease was significantly more common in CCS with CT than in CCS without CT. A recent literature review on thyroid disease after childhood cancer therapy concludes that it is unclear whether CT itself is a risk for the development of thyroid disease or whether it adds to the well-known risk of RT (38). Thyroid disorders are most frequently observed after irradiation of the neck or spinal cord (15) with the highest risk after childhood exposure (38). Due to a large number of CCS in our study population who received both RT and CT, we are unfortunately not able to differentiate our results in this regard either. Moreover, we were unable to unravel an effect of RT on the risk of thyroid diseases due to the lack of precise information from the CCS of FPN and a limited case number.

We were also able to assign the previously observed association between cancer status and dyslipidemia as therapy-related. Prolonged CT or overall reduced physical fitness due to disease and therapy were previously discussed as possible causes of such metabolic changes (18, 39). In the long term, the presence of disorders of lipid metabolism is one of the main risk factors for the development of cardiovascular diseases later in life (40, 41). Therefore, the analysis of cardiovascular outcomes after cancer therapy in childhood was particularly important to us, even if we could not observe a significant association between childhood cancer and the occurrence of cardiovascular diseases in our previously published analysis (37). In addition, in the present study, we observed no association between cardiovascular diseases and CT in childhood although such an association was reported by several other studies (17, 41, 42). As different cytostatic drugs could have different cardiotoxic effects (12, 17), the cause of this unobserved effect in our study could be the imprecision of our data. In the present study, moreover, the risk of cardiovascular diseases after RT in childhood could not be estimated due to the low number of cases. However, with regard to the latency of cardiovascular diseases and second primary malignancies, we expect an increase in these therapy-related sequalae with extended follow-up and older age.

### 4.3 Strengths and limitations

This study has several strengths. Hitherto, to the best of our knowledge, only one other validation study on retrospective assessment of cancer therapies in childhood was conducted (25). Contrary to their methods, we allowed our participants to obtain as much information as possible about previous cancer therapies before answering our self-administered questionnaire. In addition, we had access to valid information on cancer therapies from the treating hospitals of the participants as well as from therapy-optimizing studies. Therefore, this unique study sample provides the basis for the first validation of therapy information from self-administered questionnaires. The newly developed questionnaire enables in particular researchers who cannot link their study data to clinical or registry data due to infrastructural or data protection reasons to collect valid information for important research questions in the field of tumor therapy-related late sequelae in a cost-effective and efficient way. In the long term, information obtained with this questionnaire can be used to forward research on therapy-associated late effects.

However, because we used self-administered data from long-term survivors of CCS, our analysis is subject to inherent survivor bias. Severe cancer cases with high mortality, e.g., acute myeloid leukemia following acute lymphoblastic leukemia or with two consecutive cancer diagnoses in a very short time, could not be considered. Moreover, a surveillance bias cannot be excluded in our study, as former cancer patients may be diagnosed more frequently with late sequelae due to regular follow-up examinations. Since we used information from the self-reports of the participants our results might be subject to a certain recall bias, especially regarding the information on occurred adverse health effects. In addition, a selection bias cannot be ruled out and the sample size was not sufficient enough to provide enough statistical power for specific research questions, in particular regarding late adverse health effects after RT in CCS of FPN. Due to the short follow-up period and the corresponding young age of our CCS cohort, only a small number of health-related late effects have occurred so far. However, prolonged follow-up of this unique cohort of CCS and cancer-free control subjects will ensure an important and highly relevant increase in knowledge about treatment-related late effects in long-term CCS.

## 5 Conclusion

Our new self-reported questionnaire for CCS is reliable for a retrospective assessment of a general exposure to tumor therapies in childhood, particularly for CT and RT in CCS with at least one SPN. However, the self-reported information on RT provided by study participants in the FPN group was too imprecise and could not be used. Nevertheless, our questionnaire offers a simple and cost-effective way to collect valid therapy information from long-term cancer survivors. This allowed us to demonstrate an association between CT in childhood and the occurrence of some late health effects, including thyroid and lipid metabolism disorders.

## Data availability statement

The datasets presented in this article are not readily available because of ethic and data protection reasons. We are happy to support other research groups in their research projects by providing the newly developed questionnaire. Requests to access the questionnaires should be directed to the Leibniz Institute for Prevention Research and Epidemiology – BIPS, Bremen, Germany.

## Ethics statement

The studies involving human participants were reviewed and approved by the Ethik-Kommission of the Landesärztekammer Rheinland-Pfalz, Mainz, Germany. The patients/participants provided their written informed consent to participate in this study.

## Author contributions

MM is the principal investigator of the KiKme study and developed the study design and the questionnaires. The study was implemented and monitored by MM and LB. CS supported the development of strategies for the recruitment of childhood cancer survivors. MM, LB, DG, and SZ conducted the recruitment of the participants, which was organized and planned by MM and LB. MM, LB, and HSchm monitored the recruitment of controls. LB, MM, RF, and AP developed the analyses pipelines for the project. HSchw, DG, SZ, TH, AP, CS, MB, and HSchm contributed to the writing process, which was initially prepared by LB, MM, and RF. All authors contributed to the article and approved the submitted version.

## Footnotes

1
http://www.dagitty.net/dags.html.

## Supplementary material

Supplementary Figure 1 Type of self-reported cancer therapy by cancer site and year of diagnosis in participants of the nested case-control study KiKme.
