# Supplementary figures and images for: Self-administered questionnaire assessing childhood cancer treatments and associated risks for adverse health outcomes - The KiKme study
Source: Front Oncol. 2023 Apr 14;13:1150629. doi: 10.3389/fonc.2023.1150629 (PMC10147395; doi:10.3389/fonc.2023.1150629)

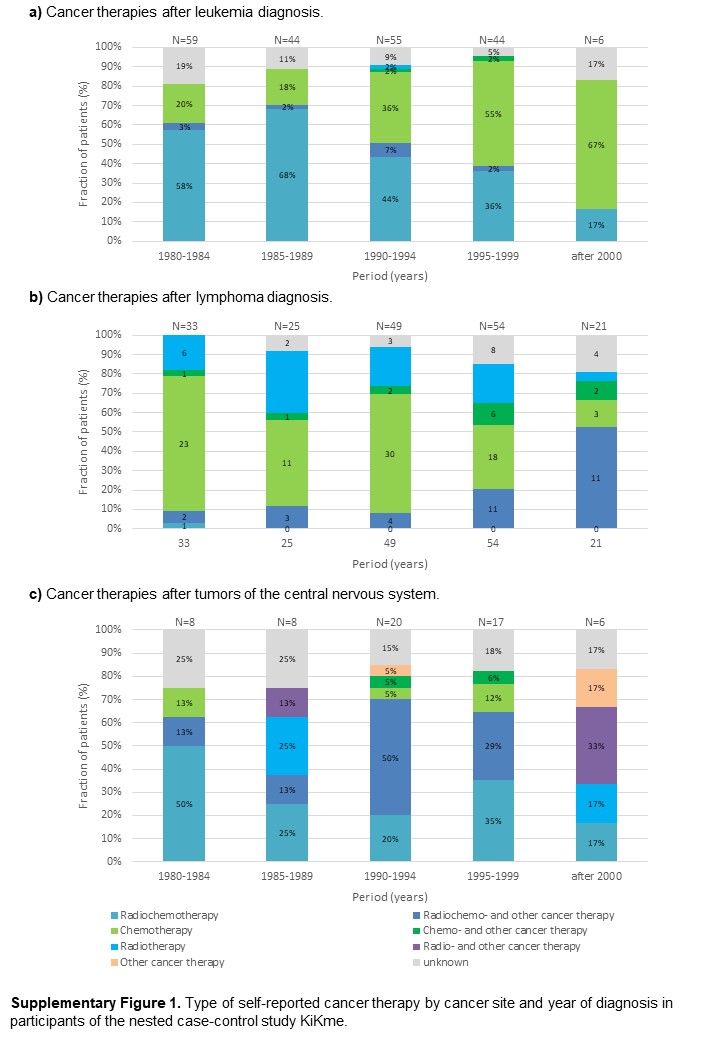

Supplement: Supplementary Figure 1 — Type of self-reported cancer therapy by cancer site and year of diagnosis in participants of the nested case-control study KiKme. [file Image_1.jpeg]
