# Supplementary material for: Self-administered questionnaire assessing childhood cancer treatments and associated risks for adverse health outcomes - The KiKme study
Source: Front Oncol. 2023 Apr 14;13:1150629. doi: 10.3389/fonc.2023.1150629 (PMC10147395; doi:10.3389/fonc.2023.1150629)
Supplement: Supplementary file 2 [file DataSheet_1.docx]

**Supplementary File 1**

**Identification of adjustment variables using Directed Acyclic Graphs (DAGs)**

**Basic adjustment:**

Matching group, age at recruitment, birth year

**Identified adjustment variables using Directed Acyclic Graphs (DAGs):**

No adjustment is necessary to estimate the total effect of therapy on health outcome.


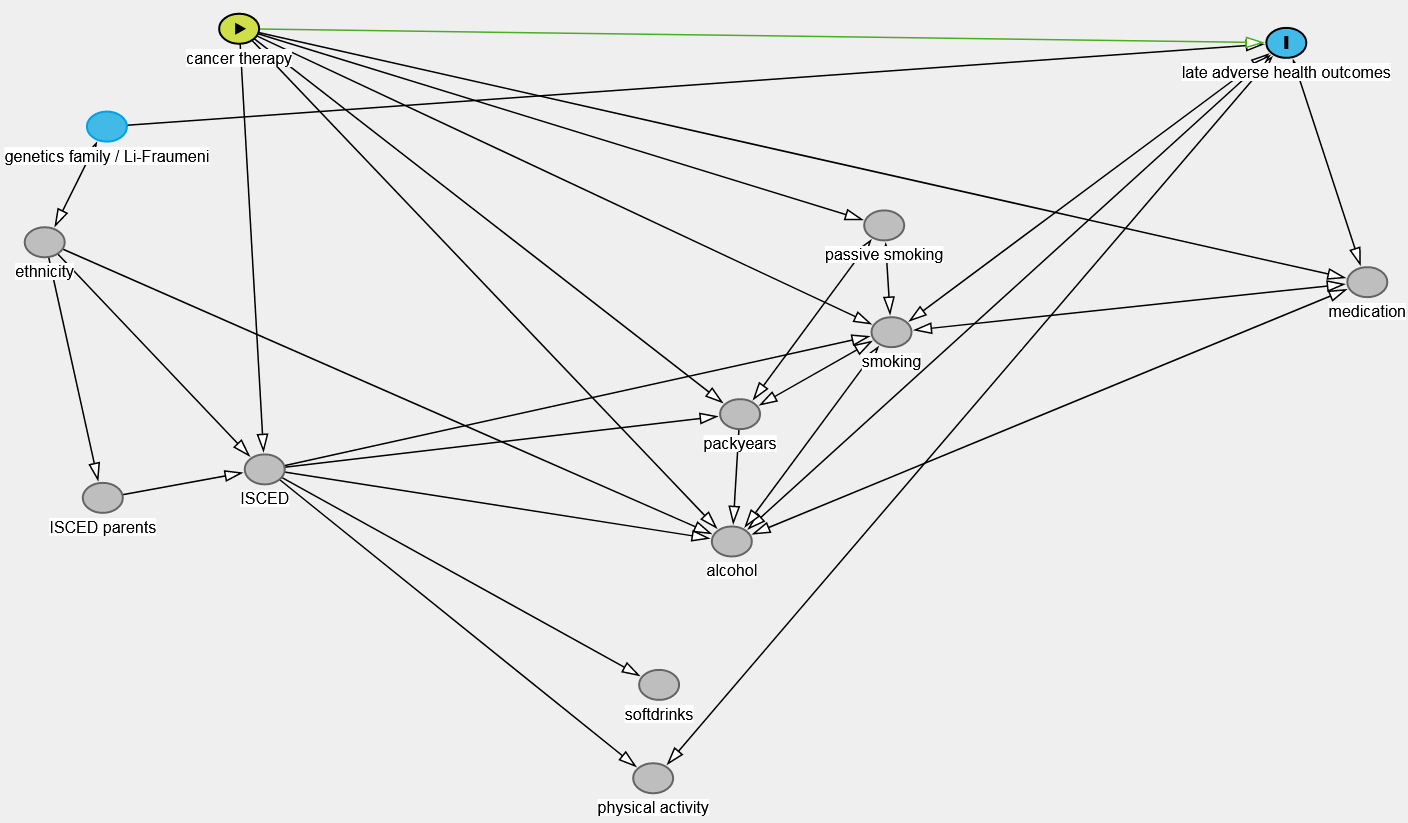


Supplementary Figure 1: DAG on the association between exposure to cancer therapies for childhood cancer and late adverse health effects.

**Code DAGs:**

dag {

"ISCED parents" [pos="-1.776,0.301"]

"cancer therapy" [exposure,pos="-1.478,-1.593"]

"genetics family / Li-Fraumeni" [pos="-1.767,-1.198"]

"late adverse health outcomes" [outcome,pos="0.811,-1.536"]

"passive smoking " [pos="-0.068,-0.799"]

"physical activity" [pos="-0.573,1.433"]

ISCED [pos="-1.422,0.186"]

alcohol [pos="-0.401,0.477"]

ethnicity [pos="-1.903,-0.731"]

medication [pos="0.988,-0.570"]

packyears [pos="-0.383,-0.037"]

smoking [pos="-0.052,-0.368"]

softdrinks [pos="-0.560,1.056"]

"ISCED parents" -> ISCED

"cancer therapy" -> "late adverse health outcomes"

"cancer therapy" -> "passive smoking "

"cancer therapy" -> ISCED

"cancer therapy" -> alcohol

"cancer therapy" -> medication

"cancer therapy" -> packyears

"cancer therapy" -> smoking

"genetics family / Li-Fraumeni" -> "late adverse health outcomes"

"genetics family / Li-Fraumeni" <-> ethnicity

"late adverse health outcomes" <-> "physical activity"

"late adverse health outcomes" <-> alcohol

"late adverse health outcomes" <-> medication

"late adverse health outcomes" <-> smoking

"passive smoking " <-> packyears

"passive smoking " <-> smoking

ISCED -> "physical activity"

ISCED -> alcohol

ISCED -> packyears

ISCED -> smoking

ISCED -> softdrinks

alcohol <-> medication

alcohol <-> smoking

ethnicity -> "ISCED parents"

ethnicity -> ISCED

ethnicity -> alcohol

medication <-> smoking

packyears -> alcohol

packyears <-> smoking

}
